# Pressure-Assisted Fabrication of Perovskite Solar Cells

**DOI:** 10.1038/s41598-020-64090-5

**Published:** 2020-04-28

**Authors:** O. V. Oyelade, O. K. Oyewole, D. O. Oyewole, S. A. Adeniji, R. Ichwani, D. M. Sanni, W. O. Soboyejo

**Affiliations:** 1 Department of Theoretical and Applied Physics, African University of Science and Technology, Km. 10 Airport Road, Galadimawa, Abuja, Federal Capital Territory Nigeria; 2 Department of Physics, Bingham University, Km. 26 Abuja-Keffi Express Way, P. M. B. 005, Karu, Nasarawa State Nigeria; 3 Department of Mechanical Engineering, Worcester Polytechnic Institute, 100 Institute Road, Worcester, MA 01609 USA; 4 Department of Physics, Baze University, Plot 686 Cadastral Zone C00, Kuchingoro Abuja, Nigeria; 5 Program in Materials Science and Engineering, Department of Mechanical Engineering, Worcester Polytechnic Institute, 100 Institute Road, Worcester, MA 01609 USA; 6 Department of Physics, Faculty of Natural and Applied Sciences, Nile University of Nigeria, Plot 681 Cadastral zone COO Research and Institution Area, Abuja, Nigeria; 7 Department of Physics, Federal University Dutsin-Ma, Dutsin-Ma, Katsina State Nigeria; 8 Department of Biomedical Engineering, Worcester Polytechnic Institute, 60 Prescott Street, Gateway Park Life Sciences and Bioengineering Center, Worcester, MA 01609 USA

**Keywords:** Materials science, Materials for energy and catalysis, Solar cells

## Abstract

This paper presents the results of a combined experimental and analytical/computational study of the effects of pressure on photoconversion efficiencies of perovskite solar cells (PSCs). First, an analytical model is used to predict the effects of pressure on interfacial contact in the multilayered structures of PSCs. The PSCs are then fabricated before applying a range of pressures to the devices to improve their interfacial surface contacts. The results show that the photoconversion efficiencies of PSCs increase by ~40%, for applied pressures between 0 and ~7 MPa. However, the photoconversion efficiencies decrease with increasing pressure beyond ~7 MPa. The implications of the results are discussed for the fabrication of efficient PSCs.

## Introduction

In recent years, organic - inorganic metal halide perovskites have been integrated into photovoltaic solar cells with power conversion efficiencies that have increased from ~3.8% to ~23.3% over a period of about 9 years^1–5^. Since these structures can be produced using low cost processing techniques^6^, this suggests that perovskite solar cells have the potential to compete with silicon solar cells that are now used in the photovoltaic industry^7^. Furthermore, since perovskite solar cells are produced at relatively low temperatures (<120 °C), a wider range of potential substrates and electrode materials can be integrated into their multilayered structures^3^. These include polymer-based flexible substrates with well adhered layers^8^, as well as transparent substrates that work well under low temperature conditions^9^. Hence, perovskite solar cells have the potential to offer the ‘golden four’ characteristics of solar cell technology. This includes: low cost, stability, efficiency and added functionality^10^.

Pressure is a unique variable that can be used to control the electronic structure and properties of organic-inorganic perovskite solar cells^11^. The application of pressure can lead to close packing, while reducing the interatomic distances. This could result ultimately in changes in the electronic orbitals and bonding patterns^12^. Hence, the application of pressure can induce changes in the structural, optical, magnetic and electronic properties^13^ of organic and inorganic solids. The application of pressure can also increase the contact between layers that are present in solar cell structures. Such contacts can suppress crack growth along the interfaces^14–19^. It can also promote charge and light transport across the interfaces of solar cells. Understanding the effect of pressure on the layers of organic-inorganic materials can enable us tune material properties through compression.

Several methods have been used to improve the optoelectronic performance of perovskite solar cells^4,5,20,21^. These include: the optimization of processing conditions^5^, the use of modifying agents^22^, and the control of starting materials^23^. Prior work^24–27^ has shown that the performance of organic electronic devices (solar cells and light emitting devices) can be improved by application of pressure. In the case of organic light emitting devices (OLEDs), the turn-on voltages of molybdenum trioxide based organic light emitting devices (OLEDs) have been significantly reduced by pressure application^24^. Improved efficiencies have also been rengineered in organic solar cells (OSCs) (from 3.5% to 4.4%) by the application of pressures of up to 10 MPa^25^. In both cases (OLEDs and OSCs), the improvements in device performance have been attributed largely to the effects of increased interfacial contacts that are associated with the application of pressure^24,25,28^. However, excessive application of pressure can also lead to the “sink in” of interlayer particles^11^, that are present at the interfaces. These can result in layer damage, and a decrease in device efficiency.

In this study, we use a combined computational/analytical and experimental approach to study the effects of pressure on the photoconversion efficiencies of multilayered perovskite solar cells. First, we use computational finite element simulations and analytical models to simulate the effects of pressure on interfacial surface contacts in the layered mixed halide PSCs. The models and simulations, which incorporates the mechanical properties of the layers in the perovskite solar cells^29,30^, show that contact between the layers increases with increasing applied pressure. The results reveal that increase pressure results in the densification of the mesoporous layers and the infiltration of the mesoporous layers with the perovskite layers.

The resulting perovskite solar cells have photoconversion efficiencies that increase from ~9.84 (9.40 ± 0.70) to 13.67 (13.10 ± 0.70) %, for pressure values between 0 and 7 MPa. However, the photoconversion efficiencies decrease with increasing pressure, for pressures beyond 7 MPa, where the increasing initial trends in the photoconversion efficiencies (p < 7 MPa), are attributed to the improved surface contacts and the initial densification and infiltration of the mesoporous layer that are associated with increasing applied pressure. However, the subsequent decrease in photoconversion efficiencies at higher pressures (p > 7 MPa) is associated with the fragmentation of the perovskite grains, and the sink-in of the perovskite layers into the mesoporous TiO_2_ layer (device damage).

## Results and Discussion

**Figure 1:**
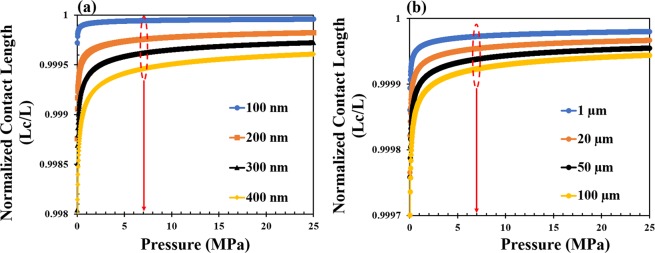
Analytical model prediction of pressure effects on contact length ratios, *L*_*C*_/*L*: (**a**) effects of pressure on the surface contacts for different thicknesses of the films (for particle size of 1 µm) and (**b**) effects of pressure on surface contacts for different sizes of the particles (for a film thickness of 250 nm).

The contact length ratios, *L*_*c*_/*L*, associated with the effects of applied pressures were obtained by the substitution of appropriate parameters into Eq. 1. First, we considered the effects of varying the thickness of the perovskite layer (100–400 nm) and the interlayer particle sizes^31^. The results of the analytical modeling of surface contact are presented in Fig. 1. For different thicknesses of the perovskite films, the interfacial surface contact length ratio *L*_*c*_/*L* increases with increasing applied pressure (Fig. 1a). The thinner films also require less pressure to wrap round the particles. This results in higher interfacial surface contacts around interlayer particles between thinner layers.

In the case where the particle sizes vary under different clean room conditions, decreasing particle sizes results in increasing interfacial surface contact (Fig. 1b).

However, upon the application of pressure, the contact lenght ratios *L*_*c*_/*L* increase with increasing applied pressure. The above results are also consistent with previous applications of Eq. 1 to organic solar cells^25,27^ and organic light emitting diodes^24^. The analytical model results suggest that increased pressure caused increased in contact between the perovskite active layer and the adjacent layers, which improves transportation of charges and work function alignment across interfaces. However, excessive pressure can lead to sink-in of the particles^15,32^, which can cause damage to the adjacent layers in perovskite solar cells. The perovskite layers can also sink into the the adjacent mesoporous layers, leading ultimately to short circuiting. Therefore, for best results, moderate intermediate pressures are required for improved contact.

Finite element modeling was also used to explore the effects of pressure on the surface contact lenght ratios *L*_*c*_/*L*, and interlayer/impurity particle sink-in. Previously obtained materials properties^29,33–36^ (Table 1) were incorporated into the finite element modeling, which was carried out using the ABAQUS software package (ABAQUS Dassault Systemes Simulia Corporation, Providence, RI, USA). The models utilized axisymmetric geometries of the device architecture. They were simplified by considering a sandwiched particle between two layers, along one of the interfaces of the device structure. The axisymmetric boundary condition was applied along the symmetry axis (Fig. S1, support information). The bottom of the substrate was also fixed to have no displacements or rotations. For continuity, the outer edge of the model was also fixed to have no lateral motion, while a pressure was applied from a stamp. The details of the finite element simulations are presented in the support information.

**Table 1:** Mechanical properties of materials used in the analytical modeling and finite element simulations. The clean room particles^14,31,37,38^ that can constitute interfacial surface void are classified along with the mechanical properties of device materials^29,33–36^.

Class	Materials	Young’s modulus (GPa)	Poisson ratio
Clean room particles	Silicone	0.001–0.02	0.3
	Photoresist	1–8	0.3
	Aluminum	70	0.3
Device Materials	FTO	206	0.32
	TiO_2_	210	0.3
	Perovskite	19.77	0.33
	Spiro-OMeTAD	15	0.36
	Au	78	0.48
	PDMS	0.003	0.3

**Figure 2:**
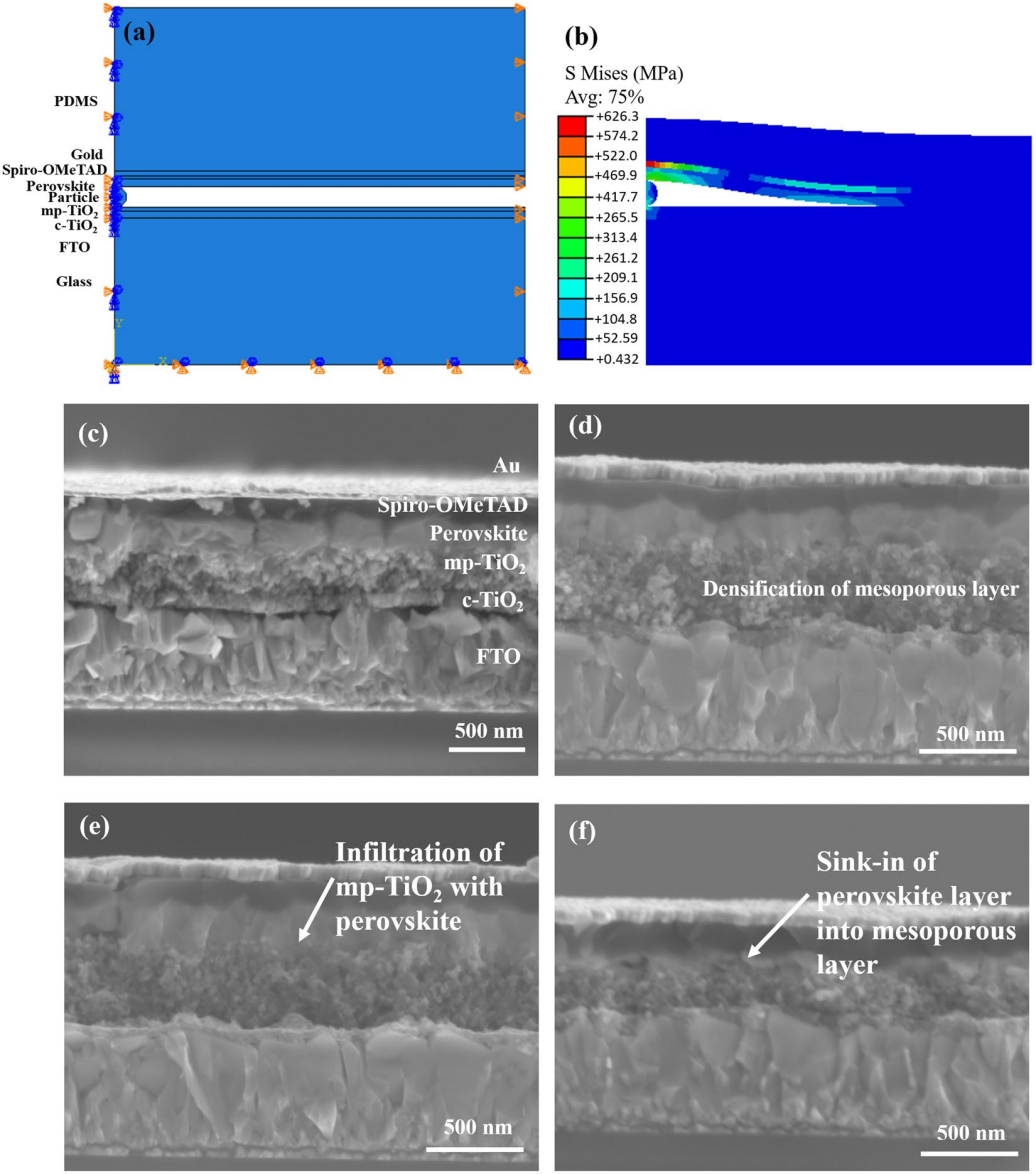
Interfacial surface contacts in perovskite solar cells before and after pressure applications: (**a**) Stress distributions before contact; (**b**) Stress distributions after contact; (**c**) Cross section of interfacial void before pressure application; (**d**) Densification of mesoporous layer after contact, (**e**) Infiltration of mesoporous structure with perovskite (p = 7 MPa) and (f) Sink-in of the perovskite layer into mesoporous and damage (p = 10 MPa).

The results of the finite element simulations (before and after pressure application) are presented in Fig. 2a,b, respectively, for the interfacial surface contact between perovskite layer and mesoporous TiO_2_ layer. Similar improvements in preesure-induced contacts were observed at other interfaces in the device structure. Furthermore, the interfacial surface contacts increased with increasing pressure (1 MPa – 10 MPa), as shown in Fig. S2a–f. Note that the effects of pressure, and the material properties of the interlayer particles on the surface contact, are presented in Fig. S3a–f. These show that the interfacial void lengths (between adjacent layers) are greatly reduced with decreasing interlayer particle moduli between 70 GPa – 0.17 GPa (for the same pressure of MPa). This range of Young’s moduli corresponds to the material properties of particles^14,31,37,38^ that are found in clean room environment (Table 1).

The above analytical and computational results are consistent with microstructural observations of the device cross sections (before and after the application of pressure), as shown in Fig. 2c–f. Note that there were significant interfacial voids between the layers of the perovskite solar cells before the application of pressure (Fig. 2c). However, the interfacial void lengths were reduced and the mesoporous layers were compacted (Fig. 2d) after the application of a pressure of 7 MPa, which resulted in the infiltration of the mesoporous TiO_2_ layers with perovskite (Fig. 2e). Sink-in of the perovskite (into the adjacent mesoporous layer) was also observed at a pressure of 10 MPa (Fig. 2f). This is consistent with the compaction and damage phenomena associated with the compressive deformation of porous materials^32^.

**Figure 3:**
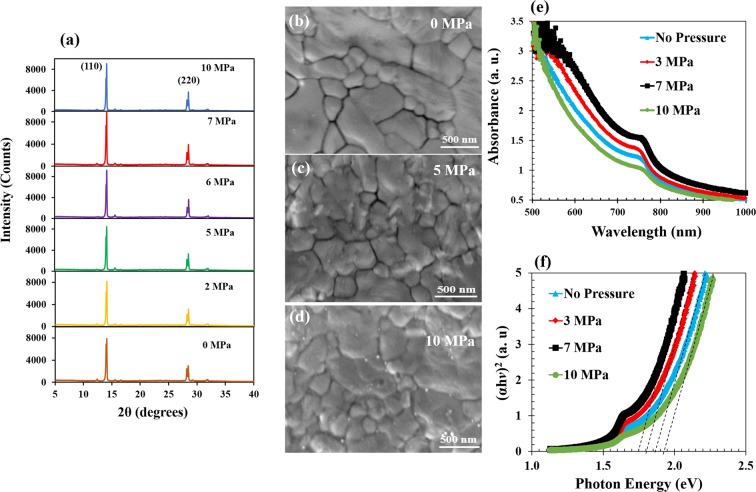
(**a–c**) XRD patterns before and after pressure application, (**d–f**) SEM images of pressure-assisted perovskite films (**g**) optical absorbance of perovskite film, and (**h**) plot of ${(\alpha hv)}^{2}$ versus photon energy.

The effects of pressure are also evident in the structural and optical properties of the perovskite solar cells. The XRD patterns of the as-prepared perovskite films and those produced via pressure-assisted fabrication are shown in Fig. 3a. It is of interest to discuss the observed effects of pressure on the crystallization of the perovskite layer. Our results show that the 110 and 220 peaks increase with increasing pressure between 0 MPa and 7 MPa. However, the peaks decrease with further increase in pressure (above 7 MPa). The SEM images of the perovskite films with the pressure-induced crystallization are shown in Fig. 3b–d. Similar observations have been reported by Wang *et al*.^39^ and Lu *et al*.^40^ who attribute the increase to crystallization phenomena due to small bond lengths that occur with increase initial pressure. Such reduction in bond lengths are also associated with stress-induced phase transformations that increase the percentage of crystalline perovskite phases with (110) and (220) orientations. However, for pressure above 7 MPa, the 110 and 220 peaks were observed to decrease with increasing pressure. The decrease is attributed to the potential onset stress-induced amorphization^39–41^ that can occur due to cracking and damage phenomena. Such localized amorphization can reduce the overall crystallinity.

These crystalline structures have also been shown by Wang *et al*.^39^, Yuan *et al*.^42^, Jiang *et al*.^41^ and Lu *et al*.^40^ to have increased absorbance due to reduction of the band gap^39–43^ in the low pressure regime. Our results show that the optical properties of the perovskite films increased with increasing applied pressure, as shown in Fig. 3e. This shows that the optical absorbance of the films increases with pressures between 0 MPa and 7 MPa due to the decrease in bond lengths. The increase in the absorbance of the perovskite film due to increased pressures is evident in the reduction bandgaps between 0 MPa and 7 MPa (as shown in Fig. 3f). For pressures above 7 MPa, the bandgaps were observed to increase with increasing pressure. This can also be attributed to local stress-induced phase changes or amorphization phenomena that can occur due to pressure application.

The bandgaps were estimated by incoporating the absorption specta into emperical formula^44^

2 $${(\alpha hv)}^{2}=hv-{E}_{g}.$$

Where *h*, *v*, *E*_*g*_ and α are Plank constant, frequency, optical energy bandgap and absorption coefficient, respectively. The decrease in the bandgap exhibits a red shift in the absorption edge^43^ that corresponds to an increase in the capacity to generate electron-hole pairs that can travel to the electrodes before recombination. It should, therefore, improve power conversion efficiencies. However, for applied pressures of 10 MPa and above, the optical absorbance decreased significantly with increasing applied pressure. High pressures can cause damage, which can lead to light scattering and unexpected blue shifts in the absorption edge.

**Figure 4:**
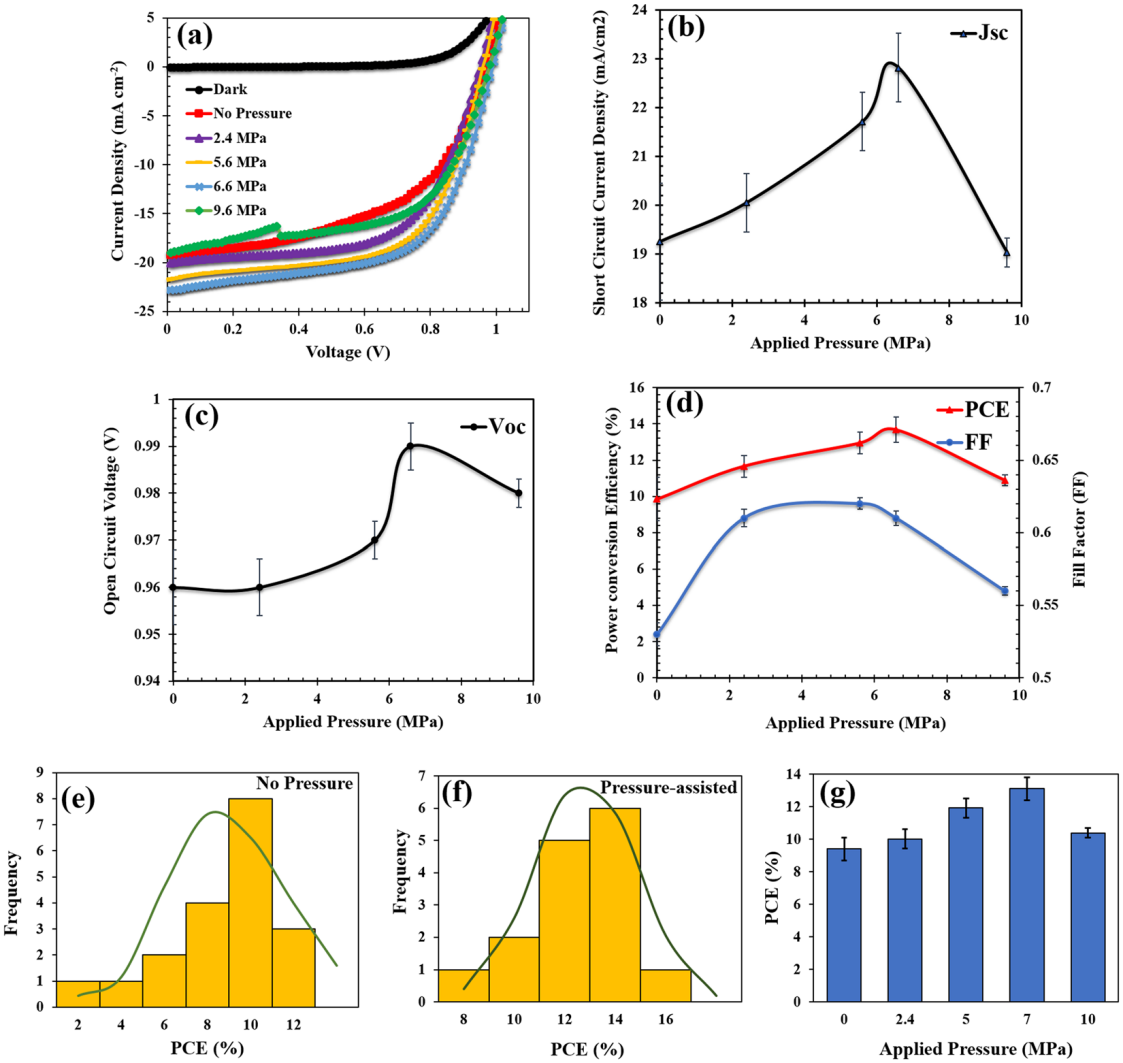
Effects of pressure on performance parameters of perovskite solar cells: (**a**) Current density-voltage curves; (**b**) short-circuit current density; (**c**) open circuit voltage; (**d**) power conversion efficiency (PCE) and fill factor for different applied pressures; (**e**) histogram and normal distribution of the PCEs of unpressurized devices (**f**) histogram and normal distribution of the PCEs of devices subjected to pressure of 2–10 MPa; (**g**) Bar chart summarizing the effects of pressure discussed in all of the fabricated devices.

The results of the device parameters (before and after pressures) are presented in Fig. 4a–g. A typical set of current density-voltage (J-V) curves obtained for the perovskite solar cells are presented in Fig. 4a. The areas under the curves increased with increasing pressure. Each of the curves is an average of J-V curves obtained from five devices. The effects of applied pressure on short circuit current density (J_sc_), open circuit voltage (V_oc_), power conversion efficiency (PCE) and fill factor (FF) are presented in Fig. 4b–d, respectively. The device characteristics are also presented in Table 2, while the overall device parameters obtained for other sets of devices are presented in Table S2.

**Table 2:** Device characteristic parameters for pressure-assisted perovskite solar cells indicating the average of the PCEs.

Pressure (MPa)	V_oc_ (V)	J_sc_ (mAcm^−2^)	FF	PCE (PCE_average_) (%)
0.0	0.96 ±0.0078	19.25 ±1.20	0.53 ±0.008	9.84 (9.40 ± 0.70)
2.4	0.96 ±0.0056	20.05 ±0.60	0.61 ±0.007	11.66 (10.01 ± 0.60)
5.0	0.97 ±0.0038	21.71 ±0.06	0.62 ±0.004	12.94 (11.92 ± 0.60)
7.0	0.99 ±0.0045	22.82 ±0.70	0.61 ±0.005	13.67 (13.10 ± 0.70)
10.0	0.98 ±0.0027	19.03 ±0.30	0.56 ±0.003	10.89 (10.02 ± 0.30)

There are two potential scenerios that could explain how relatively low applied pressures can result in high local stresses within the layered structures of perovskite solar cells. In the first scenerio, which is illustrated in Fig. 2b, one can consider the role of interfacial impurities that can give rise to interfacial stress concentration due to elastic or elastic-plastic contact. An idealized example of this is elastic-contact between spherical shapes that is often idealized by Hertzian contact theory.

**Figure 5:**
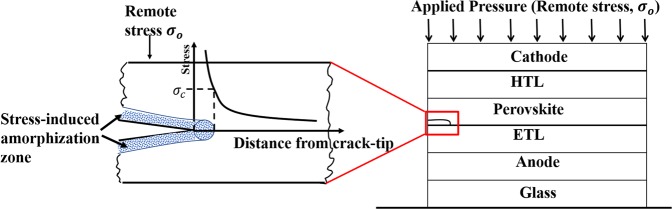
Schematic of a localized stress in an interfacial layer crack/notch within the multilayered structure of a perovskite solar cell subjected to remote pressure/stress (Effective high stresses at the crack or notch tips can induce amorphization).

The second example, which is illustrated in Fig. 5, is the case of interfacial or layer crack/notch subjected to remote stress, *σ*_0_ (as shown in Fig. 5). In such a scenerio, even under compressive loading, the induced local notch/crack stresses are much greater than the remote stresses. Furthermore, even under compressive loading, there can be induced local tensile stresses at the crack or notch tips. Such stresses may be sufficient to cause stress-induced phase changes or amorphization phenomena. Hence, it is possible to have local effects in the vicinity of such notch or crack tips that can induce phase changes/amorphization under conditions in which relatively low remote stresses are applied to a notched or cracked geometry. Based on the above arguments, the stress-induced phenomena can occur due to stress concentrations that are associated with elastic contacts around impurities and/or stress concentrations around interfacial notches or cracks.

In the case of the perovskite solar cells that were fabricated without pressure application, the PCE and FF were 9.84 (9.40 ± 0.70) % and 0.53 ± 0.008, respectively. However, with the application of pressure (up to 7 MPa), the PCE and FF increased up to 13.67 (13.10 ± 0.70) % and 0.61 ± 0.005%, respectively. For a higher applied pressure of 10 MPa, the PCE anf FF both decreased slightly to 10.89 (10.02 ± 0.30) % and 0.56 ± 0.003, respectively.

The device short circuit current density (J_sc_) and open circuit voltage (V_oc_) values (obtained at different applied pressures) increased with the applied pressures between 0–7 MPa. However, for higher applied pressures (p > 7 MPa), the performance parameters of the solar cells (J_sc_, V_oc_, FF and PCE) generally decreased (Fig. 4b–d,f).

The PCEs obtained for devices fabricated with and without pressure are summarized in the histograms (Fig. 4e,f) along with the normal distributions. The detailed histograms and normal distributions are presented in support information (Fig. S4a–e, support information). The bar chart in Fig. 4g presents a summary of the effects of pressure on PCEs of all of the 85 devices that were fabricated in this study. The results shows that the power conversion efficiencies increased with improved surface contacts at moderate pressures. However, the occurrence of interlayer particle sink-in and the compaction and damage of the mesoporous layer reduces the overall device efficiencies at higher applied pressures (Fig. 4d). Similar trends have been observed in organic solar cells^25^. However, these do not include the compaction of the mesoporous layers, which were present only in the perovskite solar cells.

**Figure 6:**
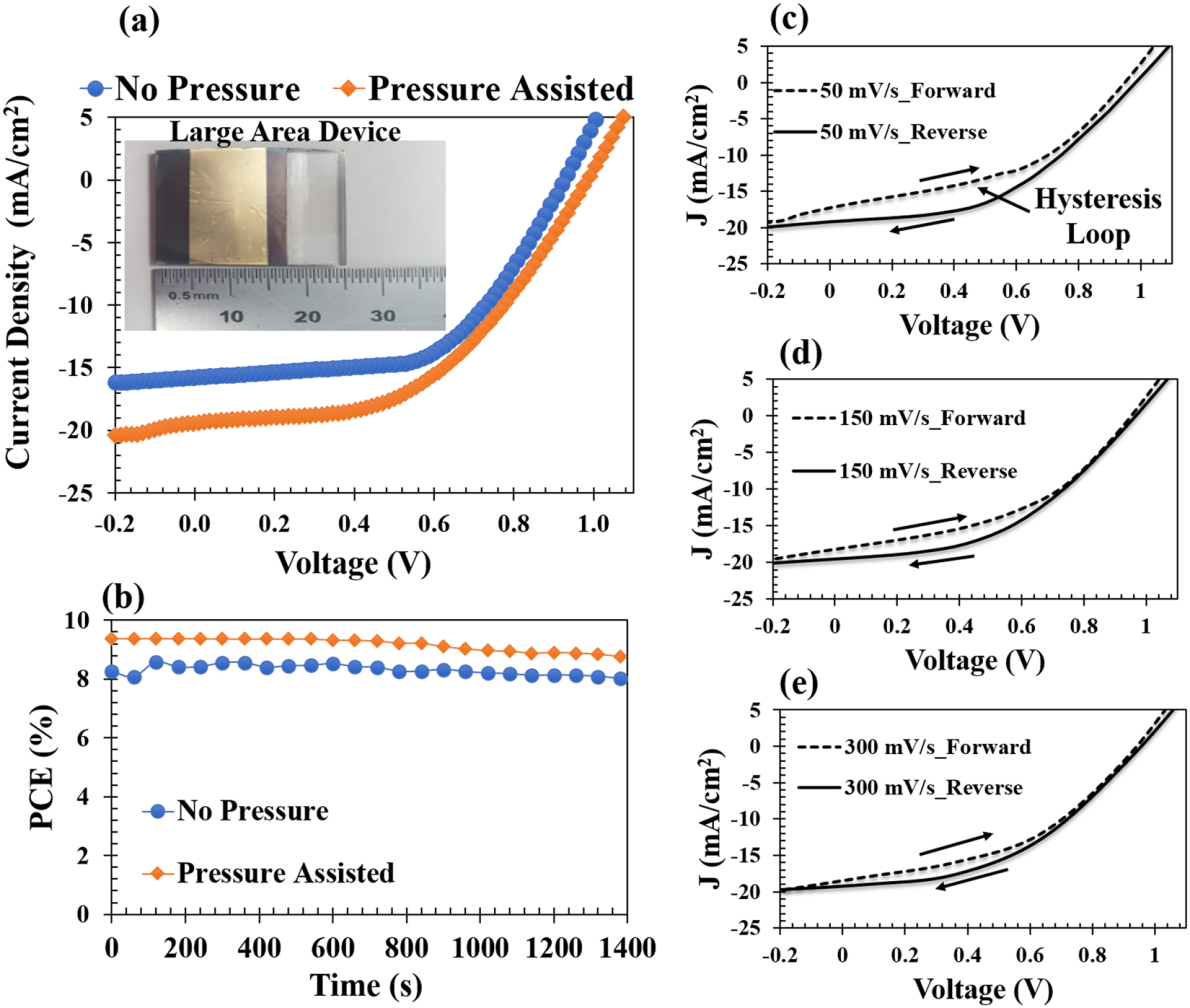
(**a**) J-V curves of pressure-assisted fabricated devices, showing the picture of the device dimension, (**b**) Steady-state PCEs of devices with large active areas under 1 sun illumination, and Hysteretic behavior of J-V curves of the devices with large active areas at scanning rates of (**c**) 50 mV/s, (**d**) 150 mV/s and (**e**) 300 mV/s.

The feasibility of the pressure-assisted fabrication technique was also demonstrated in devices with a large active area of 1.1 cm^2^ for pressure of 7 MPa. Figure 6a,b present the J-V curves and the steady-state PCEs of the large area devices under 1 sun illumination, respectively. The results showed that pressure application also enhances the PCE of the large active area devices (Fig. 6a) from 8.26 ± 0.21% to 9.38 ± 0.26%. Figure 6a inset presents the picture of one sample of the devices with large active area. The hesteretic behavior of these devices was studied at different scanning rates between 50 mV/s and 300 mV/s. Figure 6c–e present the J-V curves for both forward and reverse scanning directions at different scanning rates. The results showed that hesteresis loop decreases with increasing scanning rates (Fig. 6c–e). The dependence of hesteresis on the scanning rates and direction of the J-V curves are associated with charge carrier collection efficiencies that strongly depend on built-in potential^45^.

**Figure 7:**
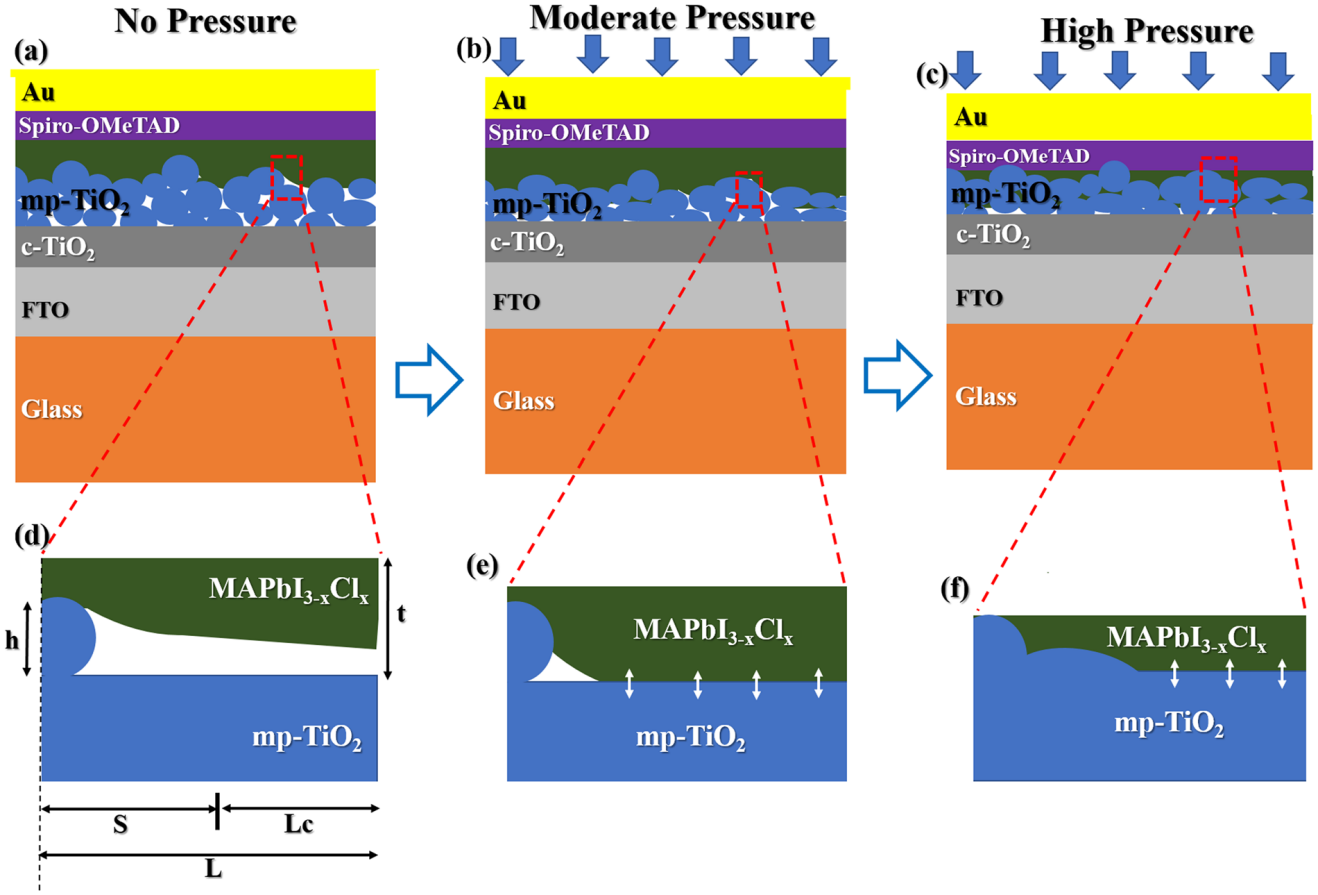
Schematics of the interfacial surface contact: (**a**) no pressure; (**b**) moderate pressure; (**c**) high pressure. Axisymmetric model of interfacial surface contact for: (**d**) no pressure case; (**e**) moderate pressure case and (**f**) high pressure.

The above results show that the power conversion efficiencies of perovskite solar cells can be significantly improved by the application of pressure. This results in the closing up of voids, and the corresponding increase in the interfacial surface contact lengths, which increases with increasing pressure (Figs. 1 and 7). Hence, the improvement in the power conversion efficiencies that was observed with increased pressure (between 0 and 7 MPa) is attributed largely to the effects of increased surface contact and the compaction and infiltration of the TiO_2_ layers with perovskite during the application of pressure.

The implications of the above results are significant for the design of pressure-assisted process that can be used for the fabrication of perovskite solar cells. First, the significant effects of pressure suggest that pressure-assisted processes such as lamination^28^, cold welding^27^ and rolling/roll-to-roll processing can be used to fabricated perovskite solar cells with improved performance characteristics (photoconversion efficiencies, fill factors, short circuit currents and open circuit voltages). However, the applied pressures should be ~7 MPa or less, to ensure that the applied pressures do not induce layer damage and the excessive sink-in of perovskite layer (between layers)^37^. Hence, the combined effects of interlayer contact, mesoporous layer compaction and infiltration and the potential for layer damage at higher pressures must be considered in the optimized design of pressure-assisted processes for the fabrication of perovskite solar cells.

## Conclusion

This paper presents the results of a combined analytical, computational and experimental study of the effects of pressure on the performance of perovskite solar cells. The results show that the application of pressure results in improved interlayer surface contact, the compaction of mesoporous TiO_2_ layers, and the infiltration of the mesoporous layers with perovskite for pressure up to 7 MPa that also result in in improved photoconversion efficiencies. However, at higher pressures (p > 7 MPa), the damage due to sink-in of the perovskite layers into the adjacent mesoporous layers resultss in reductions in the photoconversion efficiencies of perovskite solar cells.

## Methods

### Experimental methods

#### Processing of perovskite solar cells

FTO-coated glass (Sigma Aldrich) was cleaned successively in an ultrasonic bath (for 15 minutes each) in deionized water, acetone (Sigma Aldrich) and IPA (Sigma Aldrich). The cleaned glass was then blow-dried in nitrogen gas, prior to UV- Ozone cleaning (Novascan, Main Street Ames, IA, USA) for 20 minutes to remove organic residuals. Subsequently, an electron transport layer (ETL) (that comprises compact and mesoporous layers of titanium oxide) was deposited onto the FTO-coated glass. First, a compact titanium oxide (c-TiO_2_) was spin-coated onto the cleaned FTO-coated glass from a solution of titanium diisopropoxide bis (acetylacetone) (0.15 M in1-butanol) at 2000 rpm for 30 s. This was followed by 5 minutes of annealing at 150 °C before spin coating another layer of titanium diisopropoxide bis (acetylacetone) (0.3 M in 1-butanol) at 2000 rpm for 30 s. The deposited c-TiO_2_ was then annealed in a furnace (Lindberg Blue M, Thermo Fisher Scientific) at 500 °C for 30 minutes. The sample was then allowed to cool down to room-temperature (~25°C). A mesoporous titanium oxide (mp-TiO_2_) was spin coated from a solution of titanium oxide paste (20% in ethanol) at 5000 rpm for 30 s before sintering at 500 °C for 30 mins in a furnace (Lindberg Blue M, Thermo Fisher Scientific). This was then transferred into a nitrogen filled glove box, where the photoactive perovskite and the hole transport layers were deposited.

A mixed halide perovskite solution was prepared from a mixture of 222.5 mg of lead (II) iodide (PbI_2_) (>98.9% purity, Sigma Aldrich) and 381.5 mg of methylammonium chloride (MACl) (>99% purity, Sigma Aldrich) in 1 ml of dimethylformamide (DMF) (Fisher Scientific). This was then stirred at 60 °C for 6 hours in the nitrogen filled glove box. The solution was filtered using a 0.2 µm mesh filter before spin-coating onto mp-TiO_2_ at 2000 rpm for 50 s. After 30 s of the spin coating of the perovskite layer, 300 µl of chlorobenzene was then dispensed onto the film. The perovskite film was then crystallized by annealing at 90 °C for 30 minutes to crystalize. Finally, a solution of $2,{2}^{{\rm{{\prime} }}},7,{7}^{{\rm{{\prime} }}}$-tetrakis (N,N-di-p-methoxxyphenylamine)-$9,{9}^{{\rm{{\prime} }}}$-spirobifluorene (Spiro-OMeTAD) (>99% purity, Sigma Aldrich) was spin coated at 5000 rpm for 30 s.

The Spiro-OMeTAD solution was prepared from a mixture of 72 mg of Spiro-OMeTAD in 1 ml of chlorobenzene, 17.5 µl of lithium bis (trifluoromethylsulphony) imide (Li-FTSI) (Sigma Aldrich) (500 mg in 1 ml of acetonitrile), 29 µl of tris(2-(1H-pyrazol-1-yl)−4-ter-butylpyridine)-cobalt (III) tris(bis(trifluoromethaylsulfony) imide) (FK209) (Sigma Aldrich) (100 mg in 1 ml of acetonitrile) and 28.2 µl of 4-tert-butylpyridine (tBP) (Sigma Aldrich). The above film was kept overnight in a desiccator before thermally evaporating a ~ 80.0 nm thick gold (Au) layer onto the Spiro-OMeTAD from an Edward E306A evaporation system (Edward E306A, Easton PA, USA). The evaporation was carried out under a vacuum pressure of <1.0 ×10^−5^ Torr at a rate of 0.15 nm s^−1^. Shadow masks were used to define both small and large device active areas of 0.10 cm^2^ and 1.1 cm^2^ respectively. The resulting device architecture is shown in Fig. S5.

#### Pressure experiments

A range of pressures (0–10 MPa) was applied to the fabricated perovskite solar cells. This was done using a model 5848 MicroTester Instron electrochemical testing machine (Instron, Norwood, MA, USA) with a PDMS anvil placed on the device. First, the PDMS anvil was fabricated from a mixture of Sylgard 184 silicone elastomer base and Sylgard 184 silicone elastomer curing agent (Dow Corning Corporation, Midland, M I) in a ratio 10:1 by weight. The mixture was degassed and cured (at 65°C for 2 hours) in a mold with shining silicon base. The PDMS anvil was then cut out into the dimensions of the device glass substrate.

The pressure experiments are summarized schematically (Fig. S6a, support information) along with information on the Instron MicroTester set-up (Fig. S6b, support information). The Instron was set to ramp in compression at a displacement rate of 1.0 mm.min^−1^, followed by a hold at 2 MPa for 10 minutes. Unloading was then carried out at a displacement rate of ~ −1.0 mm.min^−1^. This cycle was then repeated to different peak pressures (from 2 MPa to 10 MPa) on the perovskite solar cells and perovskite layers.

#### Characterization of current density-voltage behavior

Plots of current density against voltage (J-V) were obtained for the fabricated perovskite solar cells. These were measured (before and after the pressure treatment) using a Keithley SMU2400 system (Keithley, Tektronix, Newark, NJ, USA) that was connected to an Oriel simulator (Oriel, Newport Corporation, Irvine, CA, USA) under AM1.5 G illumination of 100 mW cm^−2^. The J-V curves of devices (with zero pressure) were first measured before subsequent J-V measurements of the devices that were subjected to applied pressures of 0–10 MPa.

The optical absorbances of the as-prepared and pressure-assisted perovskite layers were measured using an Avantes UV-Vis spectrophotometer (AvaSpec-2048, Avantes, BV, USA). The X-ray diffraction patterns of as-prepared and pressure-assisted perovskite layers were also obtained using an X-ray diffractometer (Malvern PANalytical, Westborough, MA, USA). The microstructural changes of the as-prepared and pressure-assisted perovskite layers were also observed using field emission scanning electron microscope (SEM) (JEOL JSM-700F, Hollingsworth & Vose, MA, USA).

### Modeling of interfacial surface contacts due to pressure effect

The interfacial contact between the layers of perovskite solar cells is important for the

effective transportation of charges and also for work function alignment^16,38^. The integrity of the interfaces in the resulting multilayered structure also depends on the surface roughnesses of the adjacent layers^46^ and as well as the cleanliness of the environments that are used for device fabrication^47^. In particular, there are impurities/interlayer particles that can be embedded between layers in clean rooms^14,48^. These impurities include: particles of silicone, silicon, silica, textile polymer and organic materials^14,31^ with diameters ranging from ~ 0.1 to 20 µm. The presence of these particles reduces the effective contact areas (Fig. 7a) of the bi-material pairs that are relevant to the PSCs. Hence, the application of moderate pressure (to PSCs) can improve the interfacial contacts between layers that sandwich the particles (Fig. 7b). At higher pressures (Fig. 7c), however, the sink in of the trapped impurities/particles can induce damage in surrounding layers in ways that can result in reduced solar cell photoconversion efficiencies.

Hence, this section presents an analytical model for the prediction of surface contacts between layers that are relevant to PSCs. The deformation of thin films (due to applied pressure) was idealized by modeling the deformation of a cantilever beam around the particles^49–51^, as shown in Fig. 7d–f. This gives^27,28,52^:

1 $$\frac{{L}_{c}}{L}=1-{\left[\frac{3\left(\frac{E}{1-{v}^{2}}\right){t}^{3}h}{2P{L}^{4}}\right]}^{1/4},$$

where *h* is the height of the impurity particle, t is the thickness of the top layer (cantilever) that deforms upon pressure application, *S* is the void length, *L*_*c*_ is the contact length, *L* is the length of the cantilever beam, *E* is the Young’s modulus, *v* is the Poisson ratio and *P* is the applied pressure. Hence, using the materials properties of the films and particles summarized in Table 1, the interfacial surface contact lengths can be estimated for the range of pressures and film thickness and roughnesses that are relevant to the different bi-layer configurations in the multilayered perovskite solar cells structures.

## Supplementary information

Supplementary Information.
