# Supplementary material for: Pressure-Assisted Fabrication of Perovskite Solar Cells
Source: Sci Rep. 2020 Apr 28;10:7183. doi: 10.1038/s41598-020-64090-5 (PMC7188881; doi:10.1038/s41598-020-64090-5)
Supplement: Supplementary file 1 — Supplementary Information. [file 41598_2020_64090_MOESM1_ESM.docx]

Supporting Information

Pressure-Assisted Fabrication of Perovskite Solar Cells

O. V. Oyelade, O. K. Oyewole, D. O. Oyewole, S. A. Adeniji, R. Ichwani, D. M. Sanni, and W. O. Soboyejo

**1.0 Detailed Procedures of Computational Modeling**

The finite element simulations of the effects of pressure treatment were carried out using the Abaqus software package (Dassault Systemes Simulia Corporation, Providence, RI, USA). The effects of the clean room particles were considered in the simulations of contact between transport layer (TiO_2_) and the photoactive active layer (perovskite). The segments of the devices in the region of the embedded particles were analyzed in the simulations. For simplicity, axisymmetric geometries were used as shown **Fig.** S1. We assumed that the part of the device, which is farther from the dust particle, was have no significant effect on the mechanics around the dust particle. Majority of the airborne particles in semiconductor clean room environment have a diameter of $1 \mu m$,^1–4^ which is about four times of the thickness (250 - 400 nm) of the device active layer. In our simulation, a diameter of $1 \mu m$ was chosen for the dust particle. The mechanical properties of these particles are summarized in **Table** S1.

A four-node bilinear axisymmetric quadrilateral element was used in the mesh. The mesh was dense in the regions near the dust particle and the contact surfaces. Identical mesh sizes were also used in the regions near the surface contact regimes to assure convergence in contact simulation. All the materials were assumed to exhibit isotropic elastic behavior. Young’s moduli of the materials were obtained from the nanoindentation experiments as described in prior studies.^5–9^ The Young’s moduli and the Poisson’s ratios of the materials used in the simulations are summarized in **Table** S1. The axisymmetric boundary condition was applied at the symmetry axis (**Fig.** S1). The bottom of the substrate was fixed to have no displacements and rotations. The outer edge of the model was also fixed to have no lateral movement for continuity, while a pressure was applied from the stamp onto the device.


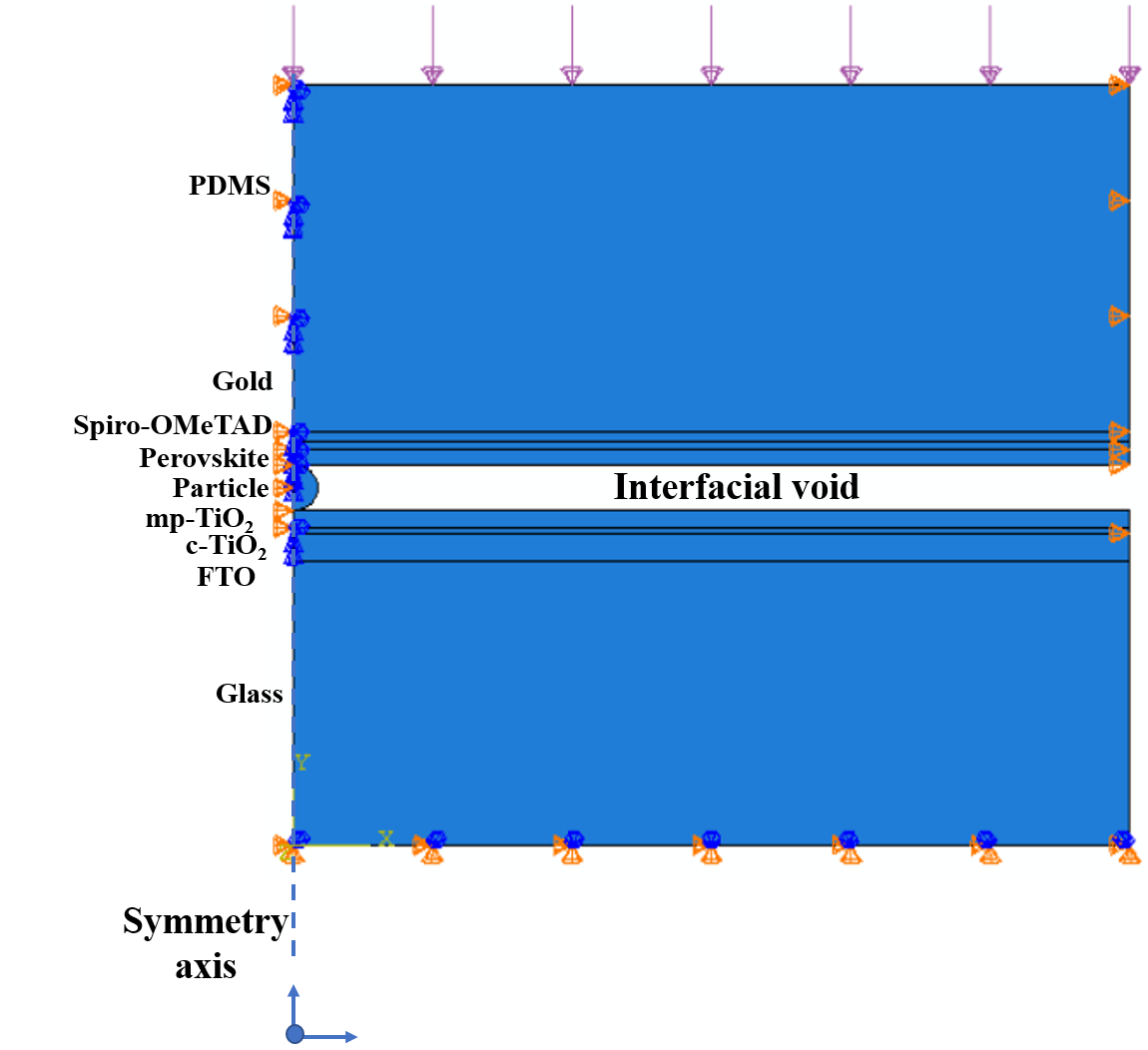


**Figure S1.** Computational modeling of the pressure-assisted perovskite solar cells, showing boundary conditions and pressure from the top.

**Table S1**. Mechanical properties of materials used in the modeling and finite element simulations. The clean room particles^1–4^ that can constitute interfacial surface void are classified along with the mechanical properties of device materials.^5–9^

| **Class** | **Materials** | **Young’s Modulus (GPa)** | **Poisson Ratio** |
| --- | --- | --- | --- |
| Clean room particles | Silicone | 0.001 – 0.02 | 0.3 |
|  | Photoresist | 1 -8 | 0.3 |
|  | Aluminum | 70 | 0.3 |
| Device Materials | FTO | 206 | 0.32 |
|  | TiO_2_ | 210 | 0.3 |
|  | Perovskite | 19.77 | 0.33 |
|  | Spiro-OMeTAD | 15 | 0.36 |
|  | Au | 78 | 0.48 |
|  | PDMS | 0.003 | 0.3 |

**2.0 Other Supporting Figures and Tables**


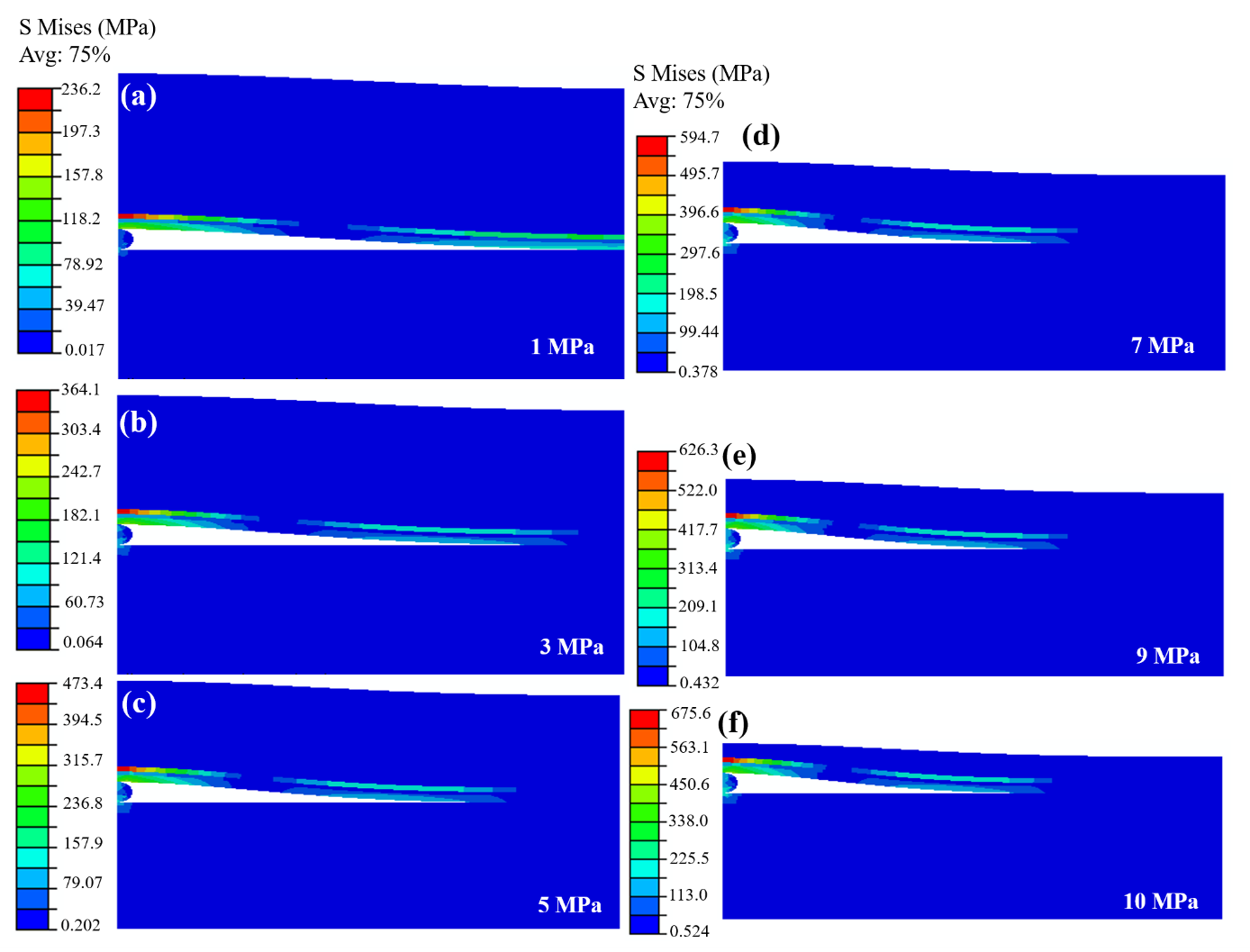


**Figure S2.** Stress distribution in perovskite solar cells during pressure application at (a) 1 MPa, (b) 3 MPa, (c) 5 MPa, (d) 7 MPa, (e) 9 MPa and (f) 10 MPa. The interfacial surface contact of the increases with increased pressure, while the interfacial void decreases. The color scale bars indicate the stress mises in MPa


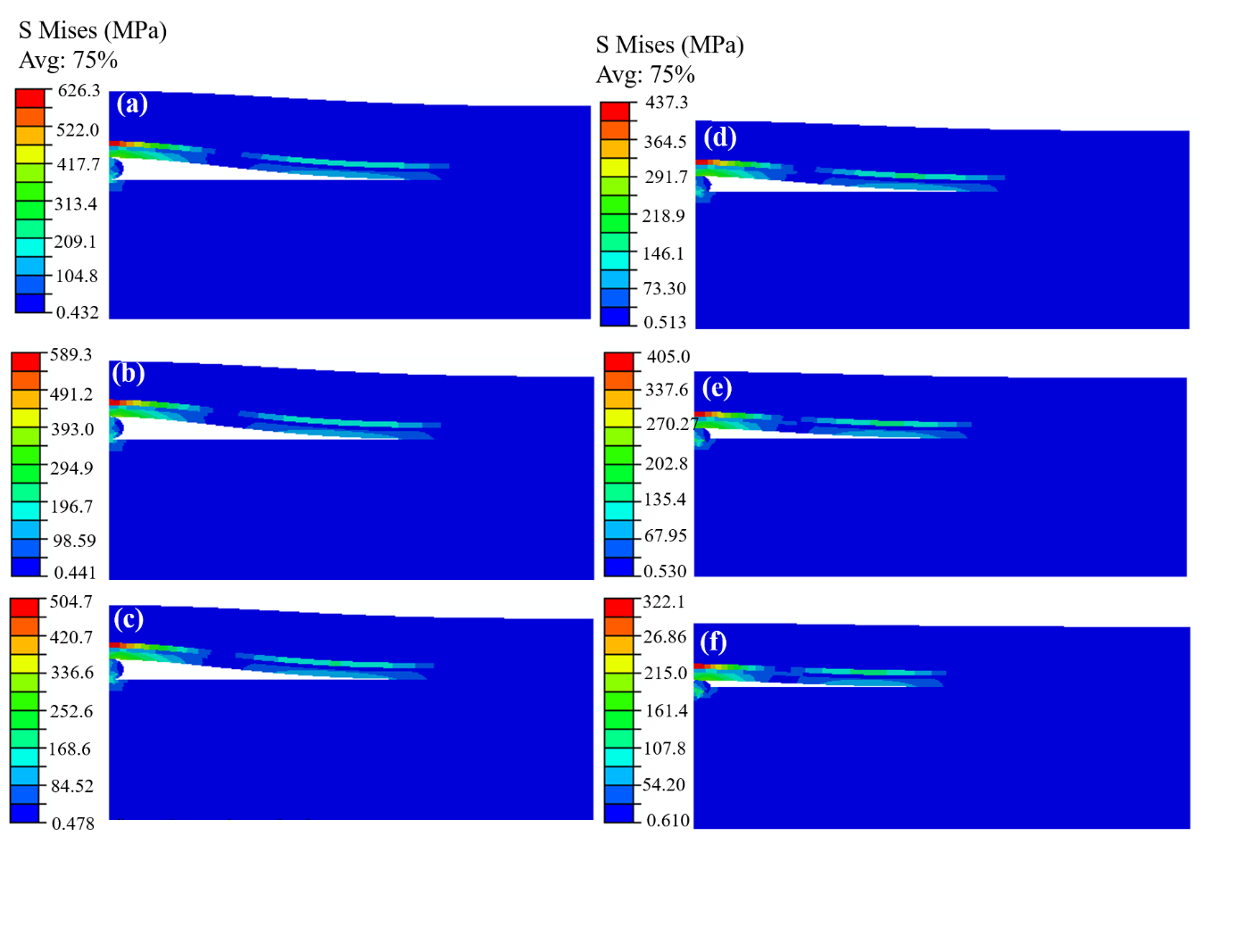


**Figure S3.** Stress distribution in perovskite solar cells during pressure application, showing effects of particles materials properties. For pressure of 9 MPa, the interfacial void reduces for particles with material properties of (a) 70 MPa, (b) 20 GPa, (c) 500 MPa, (d) 300 MPa, (e) 250 MPa and (f) 170 MPa. The color scale bars indicate the stress mises in MPa.


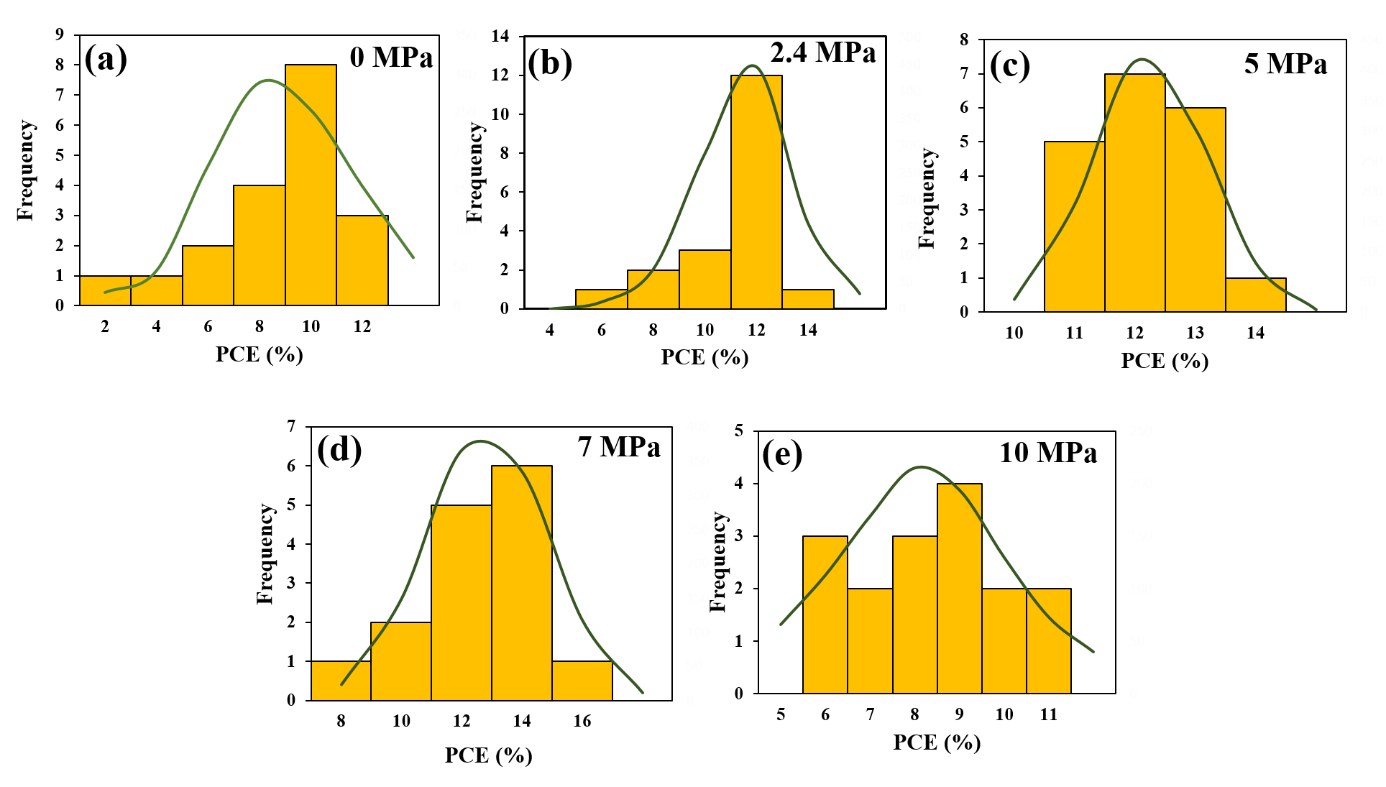


**Figure S4**. Histogram and normal distribution curves of the power conversion efficiencies of perovskite solar cells at different applied pressures (a) No pressure (0 MPa), (b) 2.4 MPa, (c) 5 MPa, (d) 7 MPa and (e) 10 MPa.


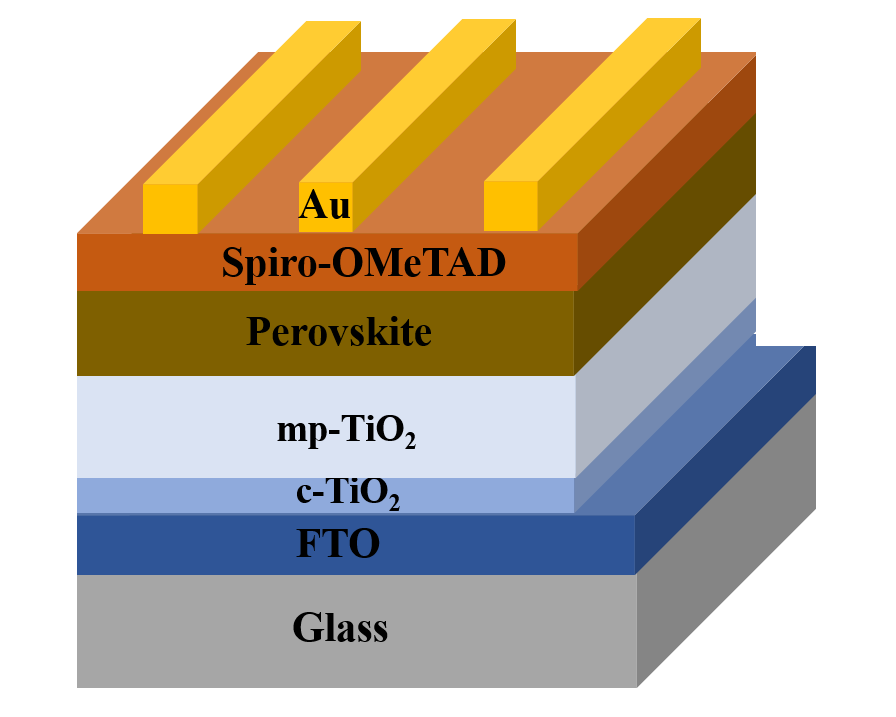


**Figure S5**. Schematic of perovskite solar cell architecture


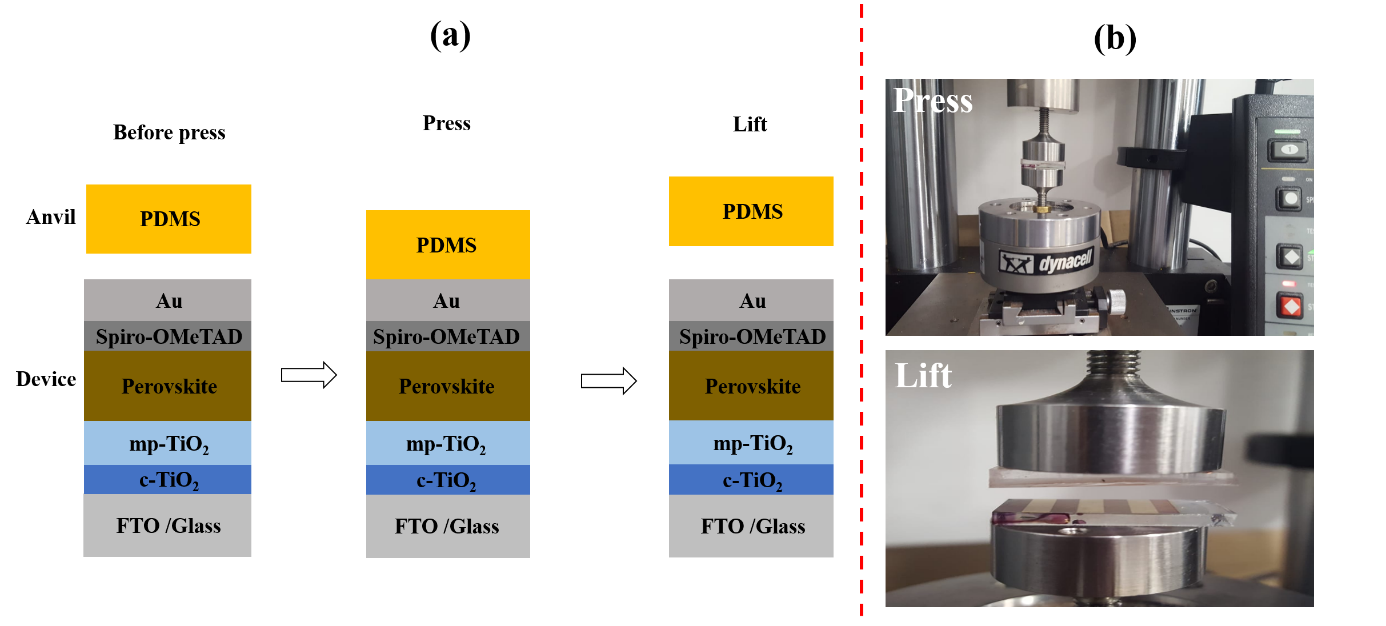


**Figure S6.** (a) Schematics of the pressure application procedures, showing before press, press and lift of the PDMS anvil. (b) Picture of the set-up of pressure application on the devices using MicroTester Instron machine for press and lift-up of the anvil.

**Table S2**. Detailed device parameters for perovskite solar cells

| Devices | Pressure  (MPa) | Voc  (V) | Jsc  (mAcm^-2^) | FF | PCE (PCE_avg_)  (%) |
| --- | --- | --- | --- | --- | --- |
| Set 1^a)^ | 0 | 0.82 | 20.88 | 0.46 | 7.91 (6.60 $\pm$ 0.90) |
|  | 2.4 | 0.92 | 21.38 | 0.54 | 10.43 (8.13 $\pm$ 0.84) |
|  | 5.6 | 0.92 | 21.44 | 0.56 | 11.58 (10.61 $\pm$ 0.74) |
| Set 2^b)^ | 0 | 0.83 | 26.64 | 0.41 | 9.22 (8.52 $\pm$ 0.61) |
|  | 2.4 | 0.83 | 27.64 | 0.40 | 9.22 (8.66 $\pm$ 0.40) |
|  | 5.6 | 0.84 | 31.85 | 0.50 | 13.22 (12.87 $\pm$ 0.48) |
|  | 7 | 0.84 | 31.72 | 0.45 | 11.65 (10.10 $\pm$ 0.49) |
|  | 10 | 0.83 | 23.87 | 0.31 | 6.22 (5.67 $\pm$ 0.52) |
| Set 3^c)^ | 0 | 0.92 | 17.51 | 0.54 | 8.72 (8.74 $\pm$ 0.40) |
|  | 2.4 | 0.92 | 19.42 | 0.61 | 10.87 (10.39 $\pm$ 0.50) |
|  | 5.6 | 0.91 | 20.14 | 0.61 | 11.23 (10.39 $\pm$ 0.90) |
|  | 7 | 0.9 | 20.11 | 0.62 | 11.12 (10.63 $\pm$0.50) |
|  | 10 | 0.92 | 16.92 | 0.47 | 7.13 (6.61 $\pm$ 0.80) |
| Set 4^d)^ | 0 | 0.96 | 19.25 | 0.53 | 9.84 (9.40 $\pm$ 0.70) |
|  | 2.4 | 0.96 | 20.05 | 0.61 | 11.66 (10.01 $\pm$ 0.60) |
|  | 5 | 0.97 | 21.71 | 0.62 | 12.94 (11.92 $\pm$ 0.60) |
|  | 7 | 0.99 | 22.82 | 0.61 | 13.67 (13.10 $\pm$ 0.70) |
|  | 10 | 0.98 | 19.03 | 0.56 | 10.89 (10.02 $\pm$ 0.30) |
| ^a)^15 devices, 5 for each applied pressure; ^b)^20 devices, 4 for each applied pressure; ^c)^25 devices, 5 for each applied pressure; ^d)^25 devices, 5 for each applied pressure; avg(average) | | | | | |

**References**

1. Cao, Y., Kim, C., Forrest, S. R. & Soboyejo, W. Effects of dust particles and layer properties on organic electronic devices fabricated by stamping. *J. Appl. Phys.* **98**, 1–6 (2005).

2. W. M. Moreau. *Semiconductor Lithography Principle, Practices and Materials Plenum*. (1988).

3. Du, J., Tong, T., Akande, W., Tsakiridou, A. & Soboyejo, W. Pressure effects on the lamination of organic light-emitting diodes. *IEEE/OSA J. Disp. Technol.* **9**, 601–606 (2013).

4. Bietsch, A. & Michel, B. Conformal contact and pattern stability of stamps used for soft lithography. *J. Appl. Phys.* **88**, 4310–4318 (2000).

5. Sun, S., Fang, Y., Kieslich, G., White, T. J. & Cheetham, A. K. Mechanical properties of organic-inorganic halide perovskites, CH3NH3PbX3 (X = I, Br and Cl), by nanoindentation. *J. Mater. Chem. A* **3**, 18450–18455 (2015).

6. Soboyejo, W. O. *Mechanical Properties of Enginneered Materials*. (Marcel Dekker Inc, 2003).

7. Tavakoli, M. M. *et al.* Efficient, flexible and mechanically robust perovskite solar cells on inverted nanocone plastic substrates. *Nanoscale* **8**, 4276–4283 (2016).

8. Park, M. *et al.* Mechanically Recoverable and Highly Efficient Perovskite Solar Cells: Investigation of Intrinsic Flexibility of Organic-Inorganic Perovskite. *Adv. Energy Mater.* **5**, 1–11 (2015).

9. Reyes-Martinez, M. A. *et al.* Time-Dependent Mechanical Response of APbX3 (A = Cs, CH3NH3; X = I, Br) Single Crystals. *Adv. Mater.* **29**, 1–7 (2017).
